# Macrophage Polarization in Left Ventricular Structural Remodeling Induced by Hypertension

**DOI:** 10.31083/j.rcm2504121

**Published:** 2024-03-28

**Authors:** Xiaolin Wu, Qiaolan Wu, Lin Gao, Yu Lv, Zhichun Wu

**Affiliations:** ^1^College of Traditional Chinese Medicine, Shandong University of Traditional Chinese Medicine, 250355 Jinan, Shandong, China; ^2^Shandong Co-Innovation Center of Classic TCM Formula, Shandong Provincial Education Department, 250355 Jinan, Shandong, China

**Keywords:** hypertension, left ventricular hypertrophy, macrophage, polarization, M1/M2 phenotype, experimental animals

## Abstract

Following long-term hypertension, mechanical stretching and neuroendocrine stimulation, cause multiple heterogeneous cells of the heart to interact, and result in myocardial remodeling with myocardial hypertrophy and fibrosis. The immune system, specifically macrophages, plays a vital role in this process. Macrophages are heterogeneous and plastic. Regulated by factors such as microenvironment and cytokines, polarization can be divided into two main forms: M1/M2, with different polarizations playing different roles in left ventricular structural remodeling associated with hypertension. However, descriptions of macrophage phenotypes in hypertension-induced myocardial hypertrophy models are not completely consistent. This article summarizes the phenotypes of macrophages in several models, aiming to assist researchers in studying macrophage phenotypes in hypertension-induced left ventricular structural remodeling models.

## 1. Introduction 

The left ventricle (LV) is the primary target of end-stage organ damage in hypertension and pressure overload-related cardiac diseases [1, 2]. The main pathological characteristics of this process involve structural remodeling, resulting in the decline of heart function, myocardial hypertrophy, and myocardial fibrosis. If left uncontrolled, this can lead to adverse events such as arrhythmias, heart failure, and death [3].

The immune system plays an important role in the process of myocardial remodeling [4], with macrophages receiving considerable attention in recent years [5]. Macrophages in the heart mainly include resident macrophages and monocyte-derived macrophages. Resident macrophages originate from fetal liver-derived macrophages and yolk sac erythro-myeloid progenitors. They continuously self-renew after birth but are gradually replaced by monocyte-derived macrophages [6].

**Fig. 1.**
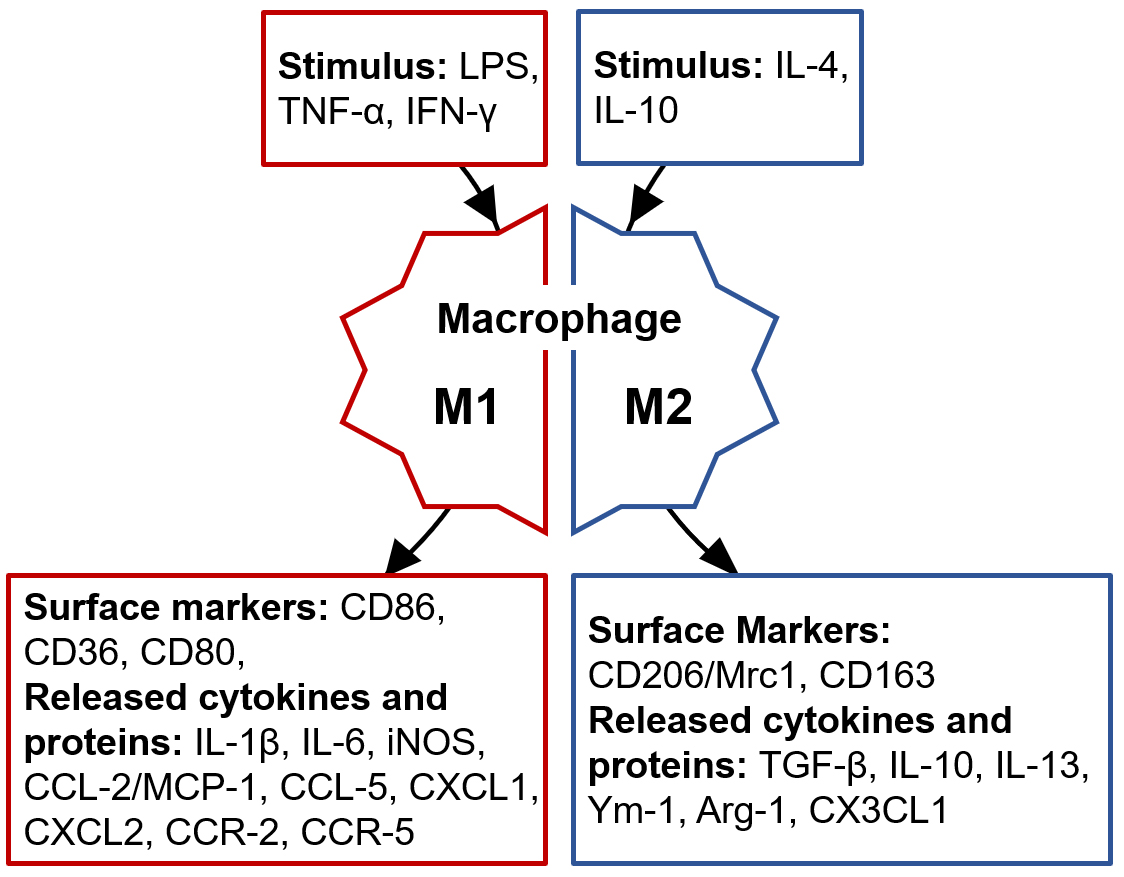
**Differing stimulus, markers, and secretions of M1/M2 macrophages phenotypes**. LPS, lipopolysaccharide; IFN-γ, interferon γ; TNF-α, tumor necrosis factor α; CD86, cluster of differentiation 86; CD36, cluster of differentiation 36; CD80, cluster of differentiation 80; IL-1β, interleukin 1β; IL-6, interleukin 6; iNOS, inducible nitric oxide synthase; CCL-2, Chemokine (C-C motif) ligand 2; MCP-1, monocyte chemotactic protein-1; CCL-5, Chemokine (C-C motif) ligand 5; CXCL1, C-X-C motif chemokine ligand 1; CXCL2, C-X-C motif chemokine ligand 2; CCR-2, chemokine receptors C-C chemokine receptor type 2; CCR-5, C-C chemokine receptor type 5; IL-4, interleukin 4; IL-10, interleukin 10; CD206, cluster of differentiation 206; Mrc1, macrophage mannose receptor 1; CD163, cluster of differentiation 163; TGF-β, transforming growth factor-beta; IL-13, interleukin 13; Ym-1, chitinase3-like1; Arg-1, arginase-1; CX3CL1, C-X3-C motif chemokine ligand 1.

In response to different stimuli, macrophages are continuously activated, ultimately resulting in two phenotypes: M1 and M2 type. M1 macrophages are classically activated, and have strong antimicrobial and anti-tumor activities. They are stimulated by pro-inflammatory substances or inflammatory factors such as tumor necrosis factor-α (TNF-α), lipopolysaccharide (LPS), and interferon γ (IFN-γ). They mediate tissue damage induced by reactive oxygen species (ROS), impede tissue regeneration and wound healing, and exert pro-inflammatory effects by releasing interleukin inflammatory mediators such as 1β (IL-1β), interleukin 6 (IL-6), ect. M2 macrophages are alternatively activated and have powerful phagocytic abilities. They are stimulated by interleukin 4 (IL-4), and interleukin 10 (IL-10). They exhibit pro-angiogenic and pro-fibrotic characteristics, exerting anti-inflammatory effects such as transforming growth factor beta (TGF-β), IL-10 ect. [7] (Fig. 1).

Macrophage polarization imbalance can lead to different pathologies in cardiac tissue. Over-polarization of M1 type promotes excessive inflammation and cardiac injury [5]. On the other hand, over-polarization of M2 type is associated with myocardial hypertrophy and extracellular matrix (ECM) expansion during the process of cardiac remodeling. In the recent literature, it has been found that the depiction of macrophage polarization phenotypes in different models of hypertension-induced myocardial hypertrophy is inconsistent. The following paragraphs will provide a brief description of several models and phenotypes.

## 2. Biochemicals-Induced Hypertension and Left Ventricular Structural Remodeling

### 2.1 Angiotensin II 

Yang *et al*. [8] found that injecting angiotensin II (Ang II) using subcutaneously implanted osmotic minipumps at a dose of 1500 ng/kg/min for 7 days in 10–12 week-old male mice led to myocardial hypertrophy, cardiac fibrosis, and inflammation. The expression of TGF-β, IL-13 and IL-10 in cardiac tissue were significantly increased, along with an increase in cluster of differentiation 206 (CD206) expression in mouse model bone marrow-derived macrophages. In addition, a member of the Protein kinase A, G, C (AGC) family of serine-threonine kinases, serum-glucocorticoid regulated kinase 1 (SGK-1), was upregulated. SGK-1 is associated with many fibrotic diseases, such as diabetic nephropathy, glomerulonephritis, pulmonary fibrosis, liver cirrhosis, and fibrotic pancreatitis. Knocking out the SGK-1 resulted in the reversal of Ang II-induced myocardial hypertrophy and cardiac fibrosis. Additionally, the expression of TGF-β, IL-13, IL-10, and CD206 in cardiac tissue were significantly decreased, and M2 macrophage polarization was blocked, suggesting that SGK-1 mediated macrophage recruitment and M2 activation. Using a three-dimensional peptide gel coculture system of bone marrow-derived macrophages and cardiac fibroblasts, it was found that SGK-1-deficient macrophages significantly reduced the activation of fibroblasts, the expression of α-smooth muscle actin (α-SMA, an active fibroblast marker), collagen I, and collagen III at the mRNA level was decreased. However, the authors did not examine the M2 marker expression in the co-culture system with SGK-1-deficient macrophages, thus the more direct relationship between SGK-1 and macrophages and between macrophage polarization and fibroblast activation, has not been demonstrated.

Myosin Heavy Chain 7 (Myh7) and Collagen 1 α 1 (Col1a1), were significantly increased in C57BL/6N mice at 10–11 weeks of age, inducted by Ang II subcutaneous osmotic pumps (500 ng/kg/day) for 48 hours or 14 days. Fibrosis-related regulating factors, tenascin (Tnc), tissue-inhibitor of metalloprotease-1 (Timp1), and lysyl-oxidase (Lox), were upregulated. The mRNA expression of osteopontin (Spp1), a marker for monocyte activation into macrophages, was significantly increased in the macrophage population isolated from the left ventricle of mice. The mRNA expression of galectin-3 (Lgals3) and Arg1, markers for M2 polarization of macrophages, was also significantly increased. These findings indicate the transition of macrophages towards an M2 phenotype [9].

A study by Reddy *et al*. [10] used subcutaneous osmotic pumps to administer Ang II (1500 ng/kg/min) to mice for 14 days. Ang II induction resulted in myocardial hypertrophy and fibrosis, with increased CD86 and CD206 positive cells in the left ventricle, indicating both anti-inflammatory and pro-inflammatory characteristics in cardiac macrophages. In this study, they found that p47phox, a subunit of nicotinamide adenine dinucleotide phosphate (NADPH) oxidase which is responsible for assembly and activation of NADPH oxidase isoform 2 (Nox2), when deficient, leads to hypertension and increases susceptibility to biomechanical stress and heart failure [11]. It regulates pressure overload-induced cardiac hypertrophy and ECM remodeling through the fibrosis signaling pathway involving signal transducer and activator of transcription 3 (STAT3) and signal transducer and activator of transcription 6 (STAT6) in macrophages, resulting in an increase in the anti-inflammatory/M2 macrophage phenotype.

Due to the association of M2 phenotype with myocardial remodeling, some authors may focus on further research on the M2 phenotype. However, this does not imply that macrophages exhibit an exclusively M2 phenotype after Ang II induction.

Kumar *et al*. [12], using osmotic pumps to infuse Ang II (980 ng/kg/min) in 8–10 weeks-old male C57BL/6J mice for 6 weeks, observed increased macrophage infiltration in cardiac tissue, along with increased expression of intercellular adhesion molecule-1 (ICAM-1). ICAM-1 is a pro-inflammatory protein that has been positively correlated with the expression of the M1 marker CD86 in inflammatory diseases such as osteoarthritis [13].

Tian *et al*. [14] found that Ang II induction in 6-week-old C57BL/6 mice (1.4 mg/kg/day) led to myocardial hypertrophy and fibrosis, along with increased expression of TGF-β1, TNF-α1, and IL-1β in cardiac tissue, indicating that both M1 and M2 phenotypes exist in induced mice. After intervention with the macrophage depleting agent Liposome encapsulated clodronate (LEC), the degree of myocardial hypertrophy and fibrosis decreased, and the expression of TGF-β1, TNF-α1, and IL-1β decreased.

### 2.2 Norepinephrine

Tang *et al*. [15] induced left ventricular hypertrophy, impaired cardiac function, decreased left ventricular systolic function, reduced ejection fraction, myocardial cell hypertrophy, and increased expression of hypertrophic genes atrial natriuretic peptide (ANP), B-type natriuretic peptide (BNP), and β-myosin heavy chain (β-MHC) by subcutaneously injecting norepinephrine (NE, 1.5 mg/kg, 0.1% ascorbic acid solution) twice daily for 15 consecutive days in C57BL/6 mice. Left ventricular myocardial tissue showed widespread expression of the macrophage marker CD68, and high expression of IL-1β, IL-6, and TNF-α in mRNA levels, indicating a tendency of macrophages towards the M1 pro-inflammatory phenotype.

Table 1 (Ref. [8, 9, 10, 12, 14, 15]) provides a summary of this section.

**Table 1.** **Different macrophage phenotypes in Ang II/NE-induced hypertensive left ventricular hypertrophy**.

Experimental animals	Age (weeks)	Induction methods	Induction dose	Duration of induction	Expression of M1 correlation factors	Expression of M2 correlation factors	Reference
B6/129S mice	10–12	Ang II	1500 ng/kg/min	7 days	-	CD206, TGF-β, IL-10, IL-13 high level	Yang *et al*. [8]
C57BL/6N	10–11	Ang II	500 ng/kg/d	48 h/14 d	-	Lgals3, Arg-1 high level	Cardin *et al*. [9]
C57BL/6 J	8–10	Ang II	1500 ng/kg/min	14 days	CD86 high level	CD206 high level	Reddy *et al*. [10]
C57BL/6J	8–10	Ang II	980 ng/kg/min	6 weeks	ICAM-1 high level	-	Kumar *et al*. [12]
C57BL/6	6	Ang II	1.4 mg/kg/d	-	IL-1β, TNF-α high level	TGF-β1 high level	Tian *et al*. [14]
C57BL/6	6–8	NE	1.5 mg/kg	15 days, twice daily	IL-1β, IL-6, TNF-α high level	-	Tang *et al*. [15]

“-” means no description provided. Ang II, injecting angiotensin II; ICAM-1, increased expression of intercellular adhesion molecule-1; IL-1β, interleukin 1β; TNF-α, tumor necrosis factor-α; IL-6, interleukin 6; TGF-β, transforming growth factor beta; IL-10, interleukin 10; Lgals3, galectin-3; Arg-1, arginase-1; NE, norepinephrine; CD206, cluster of differentiation 206; CD86, cluster of differentiation 86.

## 3. High-Salt Diet

A long-term high-salt diet can induce hypertension and myocardial hypertrophy. In animal models, mice on a high-salt diet developed left ventricular hypertrophy and reduced cardiac contractile function [16, 17].

Kain *et al*. [18] fed 6-week-old male salt-sensitive SBH/y rats a high-salt diet. After six weeks, the rats showed significantly increased systolic blood pressure. After 4 additional weeks, there was continued decline in heart function. Cardiac magnetic resonance imaging (CMR) showed increased left ventricular mass. Using flow cytometry, CD68-positive cells (macrophage marker) and markers for M1 (CD80) and M2 (CD163) phenotypes were examined. CD68 positive cells continuously increased after 6 weeks of high-salt diet intervention and reached peak levels at 10 weeks. The M2/M1 ratio was highest at 6 weeks, indicating a shift towards the M2 phenotype in macrophages. Injection of liposome-encapsulated clodronate (CL), a macrophage depleting agent, at 6 weeks of intervention resulted in smaller increases in blood pressure compared to the group not treated with the macrophage clearance agent. The macrophage clearance group also showed improved ventricular contractile function and reduced myocardial fibrosis, suggesting that in high-salt-induced cardiac remodeling, macrophages play a key role. However, the effect of macrophage depletion on blood pressure is varied and superficial among different studies [19].

Some researchers use a combined induction model of high-salt diet and other hypertensive models to better reflect the conditions for disease occurrence and ensure the development of hypertension in animals.

Yu *et al*. [17] intervened in mice for 12 weeks with a diet containing 8% NaCl or a combination of 8% NaCl diet and intraperitoneal injection of N-nitro-L-arginine methyl ester (10 mg/mL, 50 mg/kg/d). In the combined intervention group, the expression of M1 correlation factors such as TNF-α, CCL-2, IL-1β, and CCL-5, and M2 correlation factors Ym-1, Arg-1, and IL-10 were significantly increased at the mRNA level. After intervention with pseudolaric acid B, a substance with immunomodulatory effects, the expression of M1 and M2 macrophage markers showed opposite trends. TNF-α, CCL-2, IL-1β, and CCL-5 were significantly decreased after treatment, while Ym-1, Arg-1, IL-10, and macrophage mannose receptor 1 (Mrc-1) expression were significantly increased, with lower blood pressure and less LV remodeling.

Yang *et al*. [20] conducted a 4-week high-salt diet intervention with 4% NaCl in mice. They then implanted osmotic pumps to administer Ang II (2 mg/h) for 7 days. The mice exhibited a significant increase in systolic blood pressure and a significant decrease in ejection fraction and left ventricular fractional shortening. Fibrosis was observed in the myocardium, and the expression of IL-6 and monocyte chemotactic protein-1 (MCP-1) in myocardial tissue significantly increased, all of which were associated with the M1 phenotype. After knocking out IL-6, the level of myocardial fibrosis decreased, and MCP-1 expression decreased.

Table 2 (Ref. [17, 18, 20]) provides a summary of this section.

**Table 2.** **Different macrophage phenotypes in high-salt diet-induced hypertensive left ventricular hypertrophy**.

Experimental animals	Age (weeks)	Induction methods	Duration of induction (weeks)	Expression of M1 correlation factors	Expression of M2 correlation factors	Reference
SBH/y rat	6	-	10	M2 > M1 at 6th week		Kain *et al*. [18]
C57 BL/6	6	8% NaCl diet/8% NaCl diet + N-nitro-l-arginine methyl ester (10 mg/mL, 50 mg/kg/d) Intraperitoneal Injections	12	TNF-α, CCL-2, IL-1β, and CCL-5 high level	Ym-1, Arg-1, IL-10, and Mrc-1 (CD206) high level	Yu *et al*. [17]
-	4	4% NaCl diet + inject Ang II by subcutaneously implanted osmotic minipumps (2 mg/h)	4 weeks + 7 days	IL-6, MCP-1 high level	-	Yang *et al*. [20]

“-” means no description provided. TNF-α, tumor necrosis factor-α; CCL-2, Chemokine (C-C motif) ligand 2; CCL-5, Chemokine (C-C motif) ligand 5; IL-6, interleukin 6; MCP-1, monocyte chemotactic protein-1; Arg-1, arginase-1; IL-10, interleukin 10; IL-1β, interleukin 1β; Ym-1, chitinase3-like1; Mrc1, Macrophage mannose receptor 1 (as known as CD206).

## 4. Transverse Aortic Constriction Surgery

In patients with aortic stenosis, there is a higher level of infiltration of M2-type macrophages in the cardiac tissue [21]. Transverse aortic constriction (TAC) surgery simulates aortic stenosis, leading to myocardial hypertrophy, decreased cardiac contractile function, ECM collagen deposition, and cardiac fibrosis. Many studies have shown that macrophages in myocardial tissue tend to exhibit different phenotypes after TAC surgery.

Byrne *et al*. [22] performed TAC surgery on 8–10 weeks old C57BL/6 mice and found that after 5 weeks, mice showed decreased cardiac function, myocardial cell hypertrophy, myocardial fibrosis, increased expression of F4/80 (a macrophage marker), and increased expression of pro-inflammatory factors IL-6 and IL-1β. They also observed an increase in CX3CL1, which is usually driven by factors associated with M2 macrophages such as IL-10, IL-4, and Mrc-1 [23, 24].

Shen *et al*. [25] performed TAC surgery on C57BL/6J mice and found that after 4 weeks, mice showed increased expression of ANP, BNP, and β-MHC, increased myocardial cell surface area, fibrosis, increased expression of TGF-β1, increased mRNA expressions of inducible nitric oxide synthase (iNOS), CD36, CD80, and CD86 (M1 markers), and increased mRNA expressions of Arg-1, CD163, and CD206 (M2 markers).

Hackert *et al*. [26] found that expression of CCR-2, CCR-5, and C-X3-C motif chemokine receptor 1 (CX3CL1) increased in the heart, and monocytes were recruited into the heart tissue 3 days after TAC, while mRNA expression of chemotactic agents and adhesion molecules such as CX3CL1, CXCL1, and pro-inflammatory cytokine IL-1β was observed 7 weeks after TAC, indicating a shift towards the M1 phenotype in macrophages at this time point.

Methatham *et al*. [2] investigated changes in the heart tissue of mice 4 weeks after TAC. They found that 8-weeks-old male C57BL/6 mice showed impaired cardiac function, myocardial hypertrophy, increased expression of hypertrophic genes, myocardial fibrosis, and increased expression of macrophages in the myocardial tissue, as well as significantly increased expression of IL-10, IL-4, TGF-β, TNF-α, and CCL-2, indicating the coexistence of M1 and M2 macrophages in the heart tissue after 4 weeks of TAC.

In recent years, advances in single-cell RNA sequencing technology have provided a clearer understanding of disease progression. Ren *et al*. [27] divided myocardial hypertrophy after TAC into early, middle, and late stages. Based on the results of single-cell RNA sequencing, during the early stage of myocardial hypertrophy (0–2 weeks post-surgery), cardiac fibroblasts transitioned from a protective state to an activated state, which continued into the middle stage of the disease and participated in ECM expansion. Macrophages were significantly activated during the middle stage of myocardial hypertrophy (2–5 weeks post-surgery) accompanying the decline in cardiac function. These macrophages exhibited both pro-inflammatory characteristics and characteristics related to ECM organization and angiogenesis, which persisted into the late stage of myocardial hypertrophy (5–11 weeks post-surgery). Furthermore, close interaction between macrophages and fibroblasts was observed. The use of non-traditional anti-myocardial hypertrophy drugs TD139 and Arglabin to inhibit macrophage activation during this stage significantly inhibited disease progression and preserved cardiac function, while expression of myocardial fibrosis and inflammatory macrophage markers was suppressed. In the late stage of myocardial hypertrophy, macrophage subtypes associated with tissue remodeling appeared, suggesting a shift from M1 polarization to M2 polarization in macrophages.

Table 3 (Ref. [2, 22, 25, 26, 27]) provides a summary of this section.

**Table 3.** **Different macrophage phenotypes in hypertensive left ventricular hypertrophy after TAC surgery**.

Experiment-al animals	Surgery age (weeks)	Postoperative testing time	Expression of M1 correlation factors	Expression of M2 correlation factors	Reference
C57BL/6J	7–8	4 weeks	iNOS, CD36, CD80, CD86 high level	Arg-1, CD163, CD206 high level	Shen *et al*. [25]
C57BL/6	8–10	5 weeks	IL-6, IL-1β high level	CX3CL1 high level	Byrne *et al*. [22]
C57 BL/6J	-	3 days	CCR-2, CCR-5 high level	CX3CR1 high level	Hackert *et al*. [26]
		7 weeks	CXCL1, IL-1β high level	CX3CL1 high level	
C57 BL/6	8	4 weeks	TNF-α, CCL-2 high level	IL-4, IL-10, TGF-β high level	Methatham *et al*. [2]
C57 BL/6	8–10	2–5 weeks	pro-inflammatory characteristic		Ren *et al*. [27]
		5–11 weeks	subtypes associated with tissue remodeling appeared		

“-” means no description provided. iNOS, inducible nitric oxide synthase; IL-6, interleukin 6; IL-1β, interleukin 1β; CCR-2, chemokine receptors C-C chemokine receptor type 2; CCR-5, C-C chemokine receptor type 5; CXCL1, C-X-C motif chemokine ligand 1; TNF-α, tumor necrosis factor-α; Arg-1, arginase-1; CX3CL1, C-X3-C motif chemokine ligand 1; IL-4, interleukin 4; IL-10, interleukin 10; TGF-β, transforming growth factor-beta; CD36, cluster of differentiation 36; CD80, cluster of differentiation 80; CD86, cluster of differentiation 86; CD163, cluster of differentiation 163; CD206, cluster of differentiation 206; TAC, transverse aortic constriction; CX3CR1, chemokine C-X3-C motif receptor; CCL-2, Chemokine (C-C motif) ligand 2.

## 5. Genetic Hypertension Animals

### 5.1 Spontaneously Hypertensive Rats

Spontaneously hypertensive rats (SHRs) are widely recognized as a primary animal model for studying essential hypertension [28]. Over time, they develop hypertension and exhibit progressive cardiac hypertrophy, increased collagen content in the myocardium, and cardiac dysfunction due to myocardial fibrosis and interstitial remodeling.

Research has shown that at 18 weeks of age, SHRs have elevated systolic and diastolic blood pressure, as well as higher levels of IL-6 and TNF-α [29]. Another study found that at 2 months of age, SHRs have increased systolic blood pressure and elevated mRNA expressions of chemokines CXCL1 and CXCL2, which have pro-inflammatory properties. At this stage, there are no significant changes in cardiac function, myocardial cells, or extracellular matrix, but TGF-β1 levels are elevated. By 6 months of age, SHRs continue to have elevated blood pressure and exhibit increased myocardial hypertrophy, fibrosis, and inflammation, along with elevated levels of IL-6, IL-1β, TNF-α, and TGF-β1 [30].

Lee *et al*. [31] investigated the effects of human adipose-derived stem cells (hADSCs) on SHRs and found that M1 marker expression (CD68 and iNOS positive) in the SHRs myocardium was higher than after hADSC injection, while M2 marker expression (CD68 and IL-10 positive) showed the opposite trend, increasing after hADSC injection, which corresponded to reduced myocardial hypertrophy and fibrosis, as well as decreased BNP expression.

Gharraee *et al*. [32] found that M2 marker Mrc1 was expressed at a lower level in SHRs, but after intervention with a purified eicosapentaenoic acid (EPA) diet, its expression increased, promoting polarization of macrophages towards the M2 phenotype. EPA is one of the main forms of omega-3 fatty acids that have a protective effect on the cardiovascular system.

Table 4 (Ref. [29, 30, 31, 32]) provides a summary of this section.

**Table 4.** **Different macrophage phenotypes in SHRs**.

Start age (weeks)	Termination age (weeks)	Expression of M1 correlation factors	Expression of M2 correlation factors	Reference
6	18	IL-6, TNF-α high level	-	Wu *et al*. [29]
8	24	CXCL1, CXCL2 high level	TGF-β1 high level	Zhang *et al*. [30]
8	52	IL-1β, IL-6, and TNF-α high level	TGF-β1 high level	
12	20	iNOS high level	IL-10 low level	Lee *et al*. [31]
12	32	-	Mrc1 low level	Gharraee *et al*. [32]

“-” means no description provided. IL-6, interleukin 6; TNF-α, tumor necrosis factor-α; CXCL1, C-X-C motif chemokine ligand 1; CXCL2, C-X-C motif chemokine ligand 2; IL-1β, interleukin 1β; IL-6, interleukin 6; iNOS, inducible nitric oxide synthase; IL-10, interleukin 10; Mrc1, macrophage mannose receptor 1; SHRs, spontaneously hypertensive rats; TGF-β, transforming growth factor-beta.

### 5.2 Genetic Engineering

Yokono *et al*. [33] used genetic engineering techniques to insert a modified transgene into liver specific sites of apolipoprotein A1 (ApoA1) and ApoC3 in 12–16 week old mice. This resulted in sustained high expression of renin in the liver, leading to increased plasma renin activity and Ang II levels. Within 4 weeks, the mice exhibited increased systolic blood pressure, increased thickness of the interventricular septum and left ventricle posterior wall, myocardial fibrosis, increased infiltration of macrophages, and increased expression of TNF-α and TGF-β1. TNF-α and TGF-β1 are cell-signaling cytokines secreted by M1 and M2 macrophages, and play major roles in promoting inflammation and anti-inflammatory processes.

## 6. Discussion

In general, both M1 and M2 phenotypes of macrophages are present in models of hypertension-induced cardiac structural remodeling, which is accompanied by myocardial hypertrophy and activation of cardiac fibroblasts, whether stimulated by Ang II, high salt diet, or TAC surgery, or in SHRs. The specific phenotypes may vary depending on the study subjects. M2 markers in SHRs have inconsistent expression patterns across different studies, but due to limited data, no patterns have been found. Nonetheless, the relationship between M1 and M2 phenotypes in hypertensive cardiac hypertrophy is not mutually exclusive.

When studying the phenotype of macrophages, researchers investigated the changes in chemokines such as CCL-2, CCL-5, CXCL1, CXCL2, CX3CL1, and chemokine receptors such as CCR-2 and CCR-5. Most are related to M1 polarization, while CX3CL1 is related to M2-type polarization. The action of chemokines exhibit pluripotency, as they can simultaneously participate in both M1 and M2 polarization directions in macrophages [34, 35]. There is limited research on chemokines in hypertension-induced left ventricular structural remodeling, which may serve as a potential area for future studies.

It is clear that macrophages are involved in the process of LV structural remodeling in hypertension. However, further research is necessary to investigate the specific relationship between phenotypes and hypertension induced LV structural remodeling. Due to technical limitations, it is currently not possible to selectively inhibit one specific M1 or M2 phenotype of macrophages, so the exact roles of these phenotypes in LV remodeling remain unclear. Researchers can focus on specific directions for in-depth research while considering the co-expression of both phenotypes to gain a clearer understanding of the relationship between macrophages and hypertensive cardiac hypertrophy.

## 7. Conclusions

Although the exact relationship between macrophage polarization and LV structural remodeling has not been confirmed, immunotherapy may play an important role for treating LV structural remodeling in hypertension and will be the subject of future research in this area.
